# One-year psychophysical evaluation of COVID-19-induced olfactory disorders: a prospective cohort study

**DOI:** 10.1186/s12916-023-03205-x

**Published:** 2023-12-08

**Authors:** Emma J. A. Schepens, Wilbert M. Boek, Sanne Boesveldt, Robert J. Stokroos, Inge Stegeman, Digna M. A. Kamalski

**Affiliations:** 1 Department of Otorhinolaryngology- Head and Neck Surgery, University Medical Center Utrecht, P.O. Box 85500, Utrecht, 3508 GA The Netherlands; 2 Brain Center, University Medical Center Utrecht, Utrecht, The Netherlands; 3 Department of Otorhinolaryngology- Head and Neck Surgery, Hospital Gelderse Vallei, Ede, The Netherlands; 4 Division of Human Nutrition and Health, Wageningen University, Wageningen, The Netherlands

**Keywords:** Olfactory disorders, Smell, COVID-19, Prognosis

## Abstract

**Background:**

Olfactory disorders are common in COVID-19. While many patients recover within weeks, a notable number of patients suffer from prolonged olfactory disorders. Much research has focused on the acute phase of olfactory disorders in COVID-19; however, there is still inconsistency regarding the prognosis. We aim to assess both objective and subjective olfactory function in patients with persisting olfactory disorders following COVID-19, 1 year after diagnosis.

**Methods:**

We objectively measured olfactory function in 77 patients who initially had COVID-19-induced smell disorders, 1 year after confirmed diagnosis. These patients previously underwent two objective measurements at approximately 3 and 6 months after COVID-19, in the context of the COCOS trial (COrticosteroids for COvid-19-induced loss of Smell). The main outcome measurement was TDI score (threshold-discrimination-identification) on Sniffin’ Sticks Test (SST). Secondary outcomes included objective gustatory function on Taste Strip Test (TST), self-reported olfactory, gustatory and trigeminal function on a visual analogue scale (VAS) and outcomes on questionnaires about quality of life, and nasal symptoms.

**Results:**

The findings of this study show that 1 year following COVID-19, the median TDI score increased to 30.75 (IQR 27.38–33.5), regarded as normosmia. The median TDI score started at 21.25 (IQR 18.25–24.75) at baseline and increased to 27.5 (IQR 23.63–30.0) at 6 months following COVID-19. The increase of 9.5 points on the TDI score between baseline and 1 year after COVID-19 marks a clinically relevant improvement. Regarding the self-reported VAS score (1–10) on sense of smell, it increased from 1.2 (IQR 0.4–3.0) at baseline to 3.2 (IQR 1.4–6.0) at 6 months and further improved up to 6.1 (IQR 2.7–7.5) after 1 year. Objective gustatory function increased with 2 points on TST a year after diagnosis. Self-reported olfactory, gustatory, and trigeminal functions also improved over time, as did quality of life.

**Conclusions:**

Objective and self-reported olfactory function continued to improve 1 year after COVID-19. The median TDI score of 30.75 (IQR 27.38–33.5) is regarded as normosmia, which is a favorable outcome. However, the rate of improvement on TDI score reduces over time.

## Background

The importance of smell is often only recognized when it is lost. The COVID-19 pandemic has emphasized the impact of olfactory disorders, with recently reported over 50% of COVID-19 patients experience olfactory disorders [1, 2]. Although many patients have temporary olfactory disorders which resolve within weeks [2–4], according to a recent meta-analysis, about 5% of patients who initially experienced olfactory disorders will continue to have symptoms 6 months later [5].

Affected patients with persisting smell loss can suffer from decreased quality of life and malnutrition [6]. Given this significant impact olfactory disorders can have on a person’s life and the need for medical professionals to provide accurate information about recovery expectations, it is critical to increase our understanding of the clinical course of olfactory disorders after COVID-19. Although olfactory disorders in the early phase of COVID-19 have been thoroughly examined, knowledge about duration and prognosis is limited [7]. Most studies rely on self-reported sense of smell [8–18], but objective psychophysical tests can provide more precise and comparable information [19–22]. Psychological testing has been done in studies; however, comparative baseline data or a prolonged follow-up are lacking [13, 17, 23–25].

We previously reported objective improvement in a cohort of patients with COVID-19-induced persisting smell disorders (> 4 weeks) who had psychophysical measurements taken at approximately 3 and 6 months after diagnosis [26]. In the present study, we aim to determine whether this improvement in smell function is still maintained 1 year following COVID-19, by conducting a prospective cohort study with the same patients.

## Methods

### Study design

This study is a prospective cohort study and a follow-up of the COCOS trial [26, 27]. The original COCOS trial was a randomized, double-blind, placebo-controlled trial with 113 patients suffering from persisting (> 4 weeks) smell disorders within 3 months after confirmed COVID-19. Patients visited the outpatient clinic for ear, nose, and throat twice in order to measure their smell function at approximately 3 and 6 months after COVID-19 diagnosis. The first visits (baseline) were between November 2021 and February 2022. Most likely, the majority of patients was infected with the Delta variant, considering this was the dominant COVID-19 variant during that period [28]. The second visits occurred from February to May 2022. Half of the patients were treated with 40 mg oral prednisolone for 10 days. The other half received matching placebo. Researchers, physicians, and patients were blinded until after the analysis was finished. All patients were instructed and advised to perform olfactory training twice a day for at least 12 weeks. Therapy compliance was monitored by having patients fill out a daily schedule. The COCOS trial cohort (*n* = 113) performed olfactory training with median of 129 times on in 12 weeks (IQR 85–151). Both olfactory training and study treatment started the day after the first visit. Results showed no effect of prednisolone on smell function in comparison with placebo [26]. However, we found in both groups the same median improvement in all outcomes at approximately 6 months after their COVID-19 diagnosis.

**Fig. 1:**
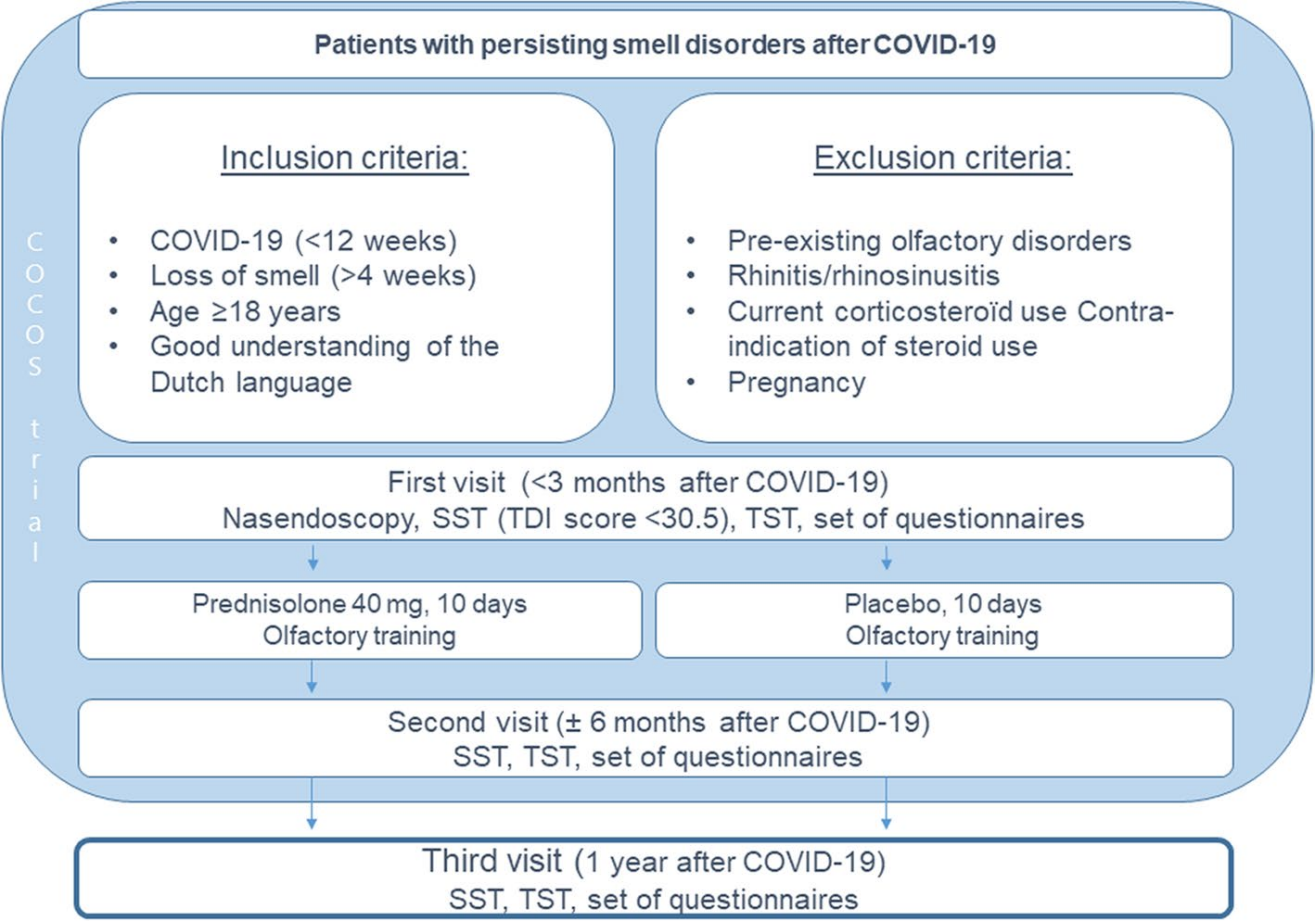
Study design. TDI score, threshold-discrimination-identification score; SST, Sniffin’ Sticks Test; TST, Taste Strip Test

For the present study, we aim to determine whether this observed improvement continues 1 year after COVID-19. To achieve this, we prolonged the follow-up period, conducting a third measurement approximately 1 year after the initial COVID-19 diagnosis (Fig. 1). Our unique advantage lay in having a cohort of patients with confirmed COVID-19 diagnosis and smell loss, with both objective and subjective measurements. We had the opportunity to conduct an additional set of measurements, enabling this subsequent prospective cohort study. This study is solely based on observing the possible progression of improvement, without any further interventions. The institutional review board of the participating hospital approved an amendment of the COCOS protocol, which allowed for this third visit (protocol number: 21–635/Gm-A) in July 2022.

### Participants

For this study, we approached participants from the COCOS trial. They were contacted by phone or email and received updated patient information forms. Results about the initial COCOS trial were already provided to the patients. The recruitment period for this study started in September 2022 and ended in January 2023. After informed consent, participants underwent the third round of measurements.

### Procedures

The third visit for the study participants took place at the outpatient ear, nose, and throat (ENT) clinic between September 2022 to January 2023. At this visit, the same smell and taste tests and questionnaires were administered as in the original COCOS trial to compare outcomes (Fig. 1). In some cases, the third measurement was conducted at the participants’ home. All measurements were recorded in an electronic case report form (eCRF) using the Castor Electronic Data Capture (EDC) system.

### Outcome measurements

The main outcome was the threshold-discrimination-identification (TDI) score on the objective Sniffin’ Sticks Test (SST). The TDI score is the sum of three different tests: a threshold (scores 0–16), discrimination (scores 1–16), and identification test (scores 1–16). The TDI score ranges from 1 to 48; a higher score is considered as a better olfactory function. Scoring ≤ 16 points is considered as anosmia, ≤ 30.5 as hyposmia, and ≤ 41.25 as normosmia. Scoring 41.5 points or above is considered as a super smeller [29]. A difference of 5.5 on TDI score was considered a clinically relevant difference [30].

Secondary objective outcome was gustatory function, measured by the Taste Strip Test (TST), which assesses recognition thresholds and identification of the four basic tastes: sweet, salty, sour, and bitter. The total score ranges from 0 to 16, with high scores indicating a better taste function. Clinical improvement was defined as a score increase of > 2 points [31].

The secondary subjective outcomes were assessed through several validated questionnaire and self-reported scales. The questionnaires included the Sino-Nasal Outcome Test-22 (SNOT-22), a visual analog scale (VAS) for self-reported smell, taste, and trigeminal sensations, and the Olfactory Disorders Questionnaire (ODQ). These questionnaires were used to measure olfactory, gustatory, and trigeminal function; the impact of smell/taste changes on quality of life; and nasal symptoms. The outcomes were assessed at the first, second, and third visits. Further details on the outcome measurements, examinations, and questionnaires can be found in the protocol, section 7.5 and 8.1.2 [27].

### Statistical analysis

The analysis was conducted using IBM SPSS Statistics 26.0.0.1. For this follow-up study, no sample size was calculated. We use descriptives to report the data. Since the data was not normally distributed, we used medians with interquartile ranges (IQR). A two-sided *P* less than 0.05 is considered a statistical significance.

## Results

### Patients

**Fig. 2:**
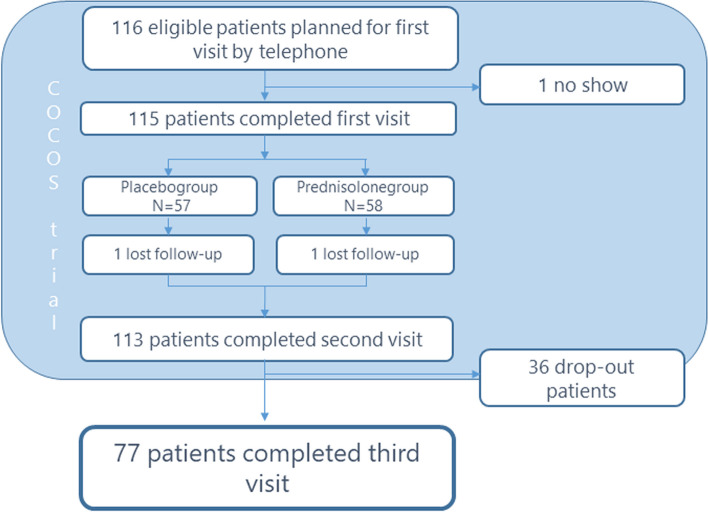
Participant flow chart

All 113 patients who completed the COCOS trial (first and second visits) were approached to participate for this follow-up study for which a third visit was required. There were 36 (31.9%) drop-outs, leading to 77 (68.1%) participating patients for this follow-up study and completing a third measurement (Fig. 2).

### Drop-out patients

Out of the original COCOS cohort, we did not conduct a third measurement at 1 year after diagnosis in 36 patients. Of these patients, 23 did not respond to the invitation to participate in this follow-up study. Additionally, three participants declined due to poor olfactory function, while one participant declined due to excellent olfactory function. Nine participants had personal reasons, such as relocation, lack of time, or physical limitations which prevented them from participating. Table 1 describes the characteristics and outcome measurements at the second visit compared between participating patients and patient drop-outs. Descriptive data at baseline, between the prednisolone and placebo arms, are described elsewhere [27]. Age and sex of both participating and drop-outs were comparable. The median age of participants in this study was 52 years old (IQR 42–59), and in the drop-out patients, it was 45.5 years old (IQR 38.8–56.5). The median TDI score on the second visit was 28.0 (IQR 23.5–30.25) in participants and 27.0 (IQR 25.38–29.69) in drop-outs. In both participating and drop-out patients, the median TST at the second visit was 11 (IQR 9–13). Scores on quality of life, self-reported smell and taste tests, and nasal symptoms seemed more favorable in patients who dropped out.

**Table 1:** Characteristics and outcome measurements at the second visit compared between participating patients and patient drop-outs. TDI score, threshold-discrimination-identification score; TST, Taste Strip Test; VAS, visual analogue scale; ODQ, Olfactory Disorders Questionnaire; SNOT-22, Sino-Nasal Outcome Test. Data are presented as median (IQR) or *n* (%). Outcome ranges were as follows: TDI 1–48; TST 0–16; VAS 0–10; ODQ 0.13–1.0; SNOT-22 0–110

	**Participating patients** ***N* = 77**	**Drop out patients** ***N* = 36**
**Age, years**	52 (42–59)	45.5 (38.8–56.5)
**Sex**		
**Female**	51 (66.2)	21 (58.3)
**Male**	26 (33.8)	15 (41.7)
**TDI score**	28.0 (23.5–30.25)	27.0 (25.38–29.69)
**TST score**	11 (9.5–13)	11 (9–13)
**VAS score**		
**Sense of smell**	2.8 (1.4–5.8)	4.35 (1.8–6.6)
**Sense of taste**	4.9 (1.6–7.2)	5.6 (3.0–8.0)
**ODQ**	0.38 (0.27–0.53)	0.37 (0.23–0.47)
**SNOT-22**	19 (10–30)	17 (8.5–3)

### Characteristics of participating patients

Table 2 describes the characteristics and outcomes during this follow-up study (third visit) and during the original COCOS trial (first and second visits) in order to compare outcomes. At the third visit, the median age was 51 years (IQR 41–58). Among 77 patients, 51 (66.2%) were female, and 26 (33.8%) were male. At least one COVID-19 vaccination was administered to 62 patients (80.5%). The median time between COVID-19 diagnosis and the third visit was 368 days (IQR 355–379). Between the second and third visits, 21 patients have had a reinfection, with mild or no complains. We did not collect specific data on the frequency and duration of olfactory training after the second visit, but 22 patients stated that they continued olfactory training on occasion.

**Table 2:** Characteristics and outcome measurements over time. Data are presented as median (IQR) or *n* (%). SNOT-22, Sino-Nasal Outcome Test; TDI score, threshold-discrimination-identification score; SST, Sniffin’ Sticks Test; TST, Taste Strip Test; ODQ, Olfactory Disorders Questionnaire; VAS, visual analogue scale. Outcome ranges were as follows: SNOT-22 0–110; TDI 1–48; T 1–16; D 0–16; I; 0–16; TST 0–16; sweet, sour, salty, bitter 0–4; ODQ 0.13–1.0; VAS 0–10

	**First visit (< 3 months after diagnosis)** ***N* = 115**	**Second visit (± 6 months after diagnosis)** ***N* = 113**	**Third visit (1 year after diagnosis)** ***N* = 77**
**Age, years**	48 (41–57)	50 (40.5–57)	51 (41–58)
**Sex**			
**Female**	73 (63.5)	72 (63.7)	51 (66.2)
**Male**	42 (36.5)	41 (36.3)	26 (33.8)
**Time from confirmed COVID test, days**	43.0 (42.5–69.5)	140 (128–154)	368 (355–379)
**Time from start of smell loss, days**	55.0 (42.0–66.8)	137 (126.3–152)	365.5 (352–376.8)
**Sino-Nasal Outcome Test (SNOT-22)**	21.0 (14.0–39.0)	18.0 (10–28)	17.0 (8.5–30.0)
**Sniffin’ Stick Test (SST)**			
**TDI score**	21.25 (18.25–24.75)	27.5 (23.63–30.0)	30.75 (27.38–33.5)
**Threshold**	1.5 (1.0–3.5)	4.5 (3.3–5.6)	6.25 (4.1–7.5)
**Discrimination**	9.0 (8.0–11.0)	11.0 (10.0–13.0)	12.0 (10.0–13.0)
**Identification**	10.0 (8.0–11.0)	11.0 (10.0–13.0)	13.0 (11.0–14.0)
**Taste Strip Test (TST)**			
**Total score**	10.0 (7.0–12.0)	11.0 (9.0–13.0)	12.0 (9.0–14.0)
**Sweet**	4.0 (2.0–4.0)	4.0 (3.0–4.0)	3.0 (3.0–4.0)
**Sour**	2.0 (1.0–3.0)	2.0 (2.0–3.0)	2.0 (2.0–3.0)
**Salty**	2.0 (1.0–3.0)	3.0 (2.0–4.0)	3.0 (2.0–4.0)
**Bitter**	2.0 (1.0–3.0)	3.0 (2.0–4.0)	3.0 (2.0–4.0)
**Olfactory Disorders Questionnaire (ODQ)**			
**Total score**	0.48 (0.41–0.57)	0.38 (0.26–0.53)	0.30 (0.21–0.43)
**Self-reported visual analogue scale (VAS)**			
**Sense of smell**	1.2 (0.4–3.0)	3.2 (1.4–6.0)	6.1 (2.7–7.5)
**Sense of taste**	3.4 (1.1–5.7)	5.3 (2.3–7.7)	7.0 (3.4–7.9)
**Trigeminal sensations**	4.5 (2.2–6.6)	5.3 (2.8–7.6)	6.10 (2.5–8.0)

### Outcomes

**Fig. 3:**
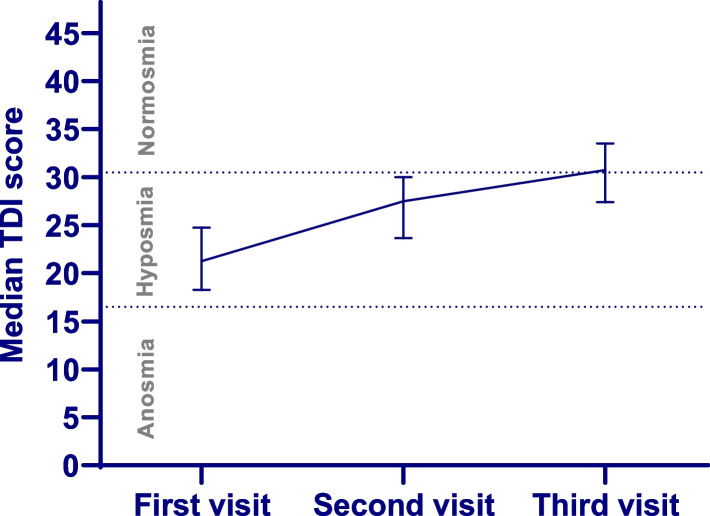
TDI scores over time

Table 2 provides outcome measurements. The median TDI score started at 21.25 (IQR 18.25–24.75) at baseline and increased to 27.5 (23.63–30.0), at 6 months after COVID-19 (Fig. 3) [26]. The findings of this study show that 1 year following COVID-19, the median TDI score was 30.75 (IQR 27.38–33.5), regarded as normosmia. The degree of improvement decreases over time; the overall improvement of 9.5 points on the TDI score exceeds the minimum clinically important difference of 5.5 points [30]. The reported VAS score (1–10) on sense of smell increased from 1.2 (IQR 0.4–3.0) at baseline to 3.2 (IQR 1.4–6.0) at 6 months to 6.1 (IQR 2.7–7.5) after 1 year.

The median score on TST 1 year after COVID-19 was 12 (IQR 9–14), starting with 10 (IQR 7–12) at baseline and 11 (9.0–13.0) at 6 months after diagnosis.

Self-reported VAS score on sense of taste also showed improvement, starting at 3.4 (IQR 1.1–5.7) and increased to 5.3 (IQR 2.3–7.7) at 6 months to 7.0 (IQR 3.4–7.9) after 1 year. The ODQ score reduced to 0.30 (IQR 0.21–0.43) 1 year after COVID-19 in comparison with 0.48 (IQR 0.41–0.57) at baseline and 0.38 (IQR 0.26–0.53) at 6 months. Nasal symptoms scored 17.0 (IQR 8.5–30.0) at SNOT-22 questionnaire and improved with 4 points in comparison with the baseline score of 21.0 (IQR 14.0–39.0). Although not the focus of our study, median TDI score for patients who received a placebo (*N* = 35, with 22 missing) was 31.5 (IQR 27.5–33.5). For patients who received the prednisolone (*N* = 41, with 15 missing), the median TDI score was 30.0 (IQR of 26.5 to 33.5).

## Discussion

We aimed to investigate the prognosis of patients with COVID-19-induced olfactory disorders. The results of this study demonstrate a favorable outcome, with a median TDI score on SST of 30.75 (IQR 27.38–33.5) 1 year after diagnosis, regarded as normosmia. Total improvement in TDI score between 3 months and 1 year after COVID-19 was 9.5 points, which exceeded the minimum clinically important difference of 5.5 points [30]. This indicates a continued recovery of olfactory function, even after a prolonged time. We already knew improvement on smell function occurs during the initial period following COVID-19 [26]. This study showed a persisting recovery process a year after diagnosis, albeit with deceleration over time. While the recovery to normosmia seems promising, it does not necessarily mean that every individual will regain their pre-COVID-19 sense of smell.

Our secondary outcomes demonstrated ongoing improvement as well. This included continued improvement on objective gustatory function on TST as well as improvements in self-reported quality of life and sense of smell and taste. The favorable outcome observed in our study is likely due to a natural course of recovery, although it is also possible that the olfactory training our cohort performed in the early phase after COVID-19 may have had a positive effect.

Other studies using psychophysical tests lack long-term follow-up [13, 23], and studies that do have long-term follow-up lack psychophysical measurements [9–12, 14–16, 32]. There have been studies with psychophysical tests performed a long time after COVID-19, however without comparative baseline data. One study utilized the psychophysical University of Pennsylvania Scent Identification Test (UPSIT) 1 year after COVID-19, which had a median score of 31 (IQR = 5.0) [17]. This is categorized as mild microsmia in the UPSIT score [33]. Another case–control study conducted a SST at 401 days after COVID-19, showing in a median TDI score of 31.5 [24]. Our TDI scores 1 year after COVD-19 fit within the range of these TDI scores. Two other studies performed the SST from 1 up to 2 years after COVID-19 and found that, while some individuals continued to recover over time, others still exhibited olfactory disorders after 2 years [25, 32]. Finally, one study compared olfactory disorders between patients in the first and second waves of COVID-19 using extended SST at various time points after infection and found just like our results that most recovery occurred in the early stage after COVID-19 [34].

While these studies contribute to our understanding of the course of olfactory disorders following COVID-19, they either lack comparable baseline data or extended psychological tests or the patients included did not initially suffer from objective olfactory disorders after COVID-19. As a result, their study designs are not intended to observe the course of olfactory function in patients with olfactory disorders over time.

With this study, we present the 1-year results of a cohort with COVID-19-induced olfactory disorders. We conducted psychophysical testing at baseline, intermediate, and 1 year follow-up stages of COVID-19. This allows for a comparative analysis of TDI scores over time, providing objective data on improvement. We used a standardized protocol, combining objective and self-reported subjective data, thereby covering all outcomes important for assessing the course of COVID-19-induced olfactory disorders. Besides, our study was solely focused on patients with smell disorders following COVID-19. This ensured that patient participation was not biased towards any other particular post-COVID-19 syndrome.

Additionally, all patients had a confirmed COVID-19 diagnosis, and follow-up period was prolonged.

There are however some restrictions of our study to take in account, the most important one being that the 1-year measurement was not foreseen in the initial set-up of the study; therefore, the outcomes were not taken into account in the first manuscript [26]. This might also reflect in the participation numbers to the 1-year measurement. Due to some patients’ dropouts, there may be some possible patient selection bias in this follow-up study. We were unable to determine for all of the drop-out patients their reasons for declining to participate in this follow-up study, though, as reported, drop-outs and participants were comparable in characteristics. The study’s generalizability could also be restricted as it solely included participants from the Netherlands. Besides, the inquiry into the impact of COVID-19 reinfections on the recovery process is an intriguing question. We inquired with our participants regarding COVID-19 reinfections, but the results in the third measurement phase were unreliable due to inconsistent testing practices and reduced testing needs. Secondly, because of the timing of our study, patients were mainly infected by the Delta variant. COVID-19 variants and vaccination status might influence the speed and extent of recovery. Thirdly, during the cohort recruitment phase, the prevailing knowledge suggested that most patients spontaneously recover their normal smell and taste function within 4 weeks [35]. Therefore, intervening during this period carried the risk of overtreatment. Additionally, the use of prednisolone to manage COVID-19 could potentially inhibit the immune system and prolong the infection. In light of our current knowledge, we might have considered enrolling participants with a more extended duration of persistent smell loss as eligibility criteria [9, 36].

Lastly, it should be noted that approximately half of the participants in our follow-up study was earlier treated with 10 days of 40 mg oral prednisolone in the context of the COCOS trial. Since no effect of prednisolone was shown on olfactory function, it is unlikely to have influenced our outcomes.

In terms of clinical implications, patients could be reassured by the findings of our study, which indicate a continued recovery of olfactory function after a year, albeit at a slower rate over time. Not only objective results are favorable, quality of life and self-reported smell function improved as well. Despite this promising news for patients, healthcare workers face a challenge due to the large numbers of patients suffering from olfactory disorders with limited treatment options. Considering the findings of McWilliams [11], which showed self-reported sense of smell 2 years after COVID-19 with 7.5% reporting no recovery, future research may further prolong follow-up period with psychophysical tests. Above that, the potential impact of olfactory training on the observed improvements in this cohort warrants further investigation.

## Conclusions

Our study demonstrated a continued recovery process on COVID-19-induced olfactory disorders, 1 year after diagnosis. The median objective smell function surpassed the threshold of normosmia, and the rate of improvement between baseline and the 1-year measurement was considered as clinically relevant. However, the rate of improvement reduces over time. Aside from smell function, objective gustatory function on Taste Strip Tests continued to improve after a year, as did self-reported quality of life and sense of smell and taste function.

## Data Availability

Participant data are available from the corresponding author under reasonable request.

## Abbreviations

CI: Confidence interval
COCOS: Corticosteroids for COVID-19-induced smell loss
eCRF: Electronic case report form
EDC: Electronic Data Capture
ENT: Ear, nose, and throat
IBM SPSS: IBM software for Statistical Package for the Social Sciences
IQR: Interquartile range
ODQ: Olfactory Disorders Questionnaire
PCR: Polymerase chain reaction
SD: Standard deviation
SNOT-22: Sino-Nasal Outcome Test-22
SST: Sniffin’ Sticks Test
TDI: Threshold-discrimination-identification
TST: Taste Strip Test
VAS: Visual analogue scale
